# Aspirations of Gas and Fluid in Strangulated Hernia

**Published:** 1872-01

**Authors:** 


Aspiration of Gas and Fluid in Strangulated Hernia.—M. Duplouy, Professor of Clinical Surgery at Rochefort, (*Gaz. Hebd.*, February 6th, 1871,) having been consulted in a case of strangulated hernia of three days' standing, in a man of eighty-two, had recourse to the above-mentioned operation. Herniotomy was not thought advisable on account of the patient's age, and the needle and aspirating syringe were used. Only gas was at first evacuated, and, by continuing the play of the piston, fluid tinged with fæcal matter was obtained. The bowel thereupon became flaccid, and was easily returned. The case did well, and it is supposed that, the puncture being very fine, the fibres closed the minute aperture by lying against each other, fatal effusion being thus prevented.

Evacuation of gas by puncturing has been in vogue of late with French surgeons in cases of tympanitis, the result being in some instances favorable. Dr. Rahn (*Montpelier Med.*, June, 1871,) succeeded in a case of strangulated hernia by injecting in the vicinity of the tumour the twenty-fifth part of a grain of sulphate of atropine.

